# Artificial intelligence guided enhancement of digital PET: scans as fast as CT?

**DOI:** 10.1007/s00259-022-05901-x

**Published:** 2022-07-29

**Authors:** René Hosch, Manuel Weber, Miriam Sraieb, Nils Flaschel, Johannes Haubold, Moon-Sung Kim, Lale Umutlu, Jens Kleesiek, Ken Herrmann, Felix Nensa, Christoph Rischpler, Sven Koitka, Robert Seifert, David Kersting

**Affiliations:** 1 Institute of Diagnostic and Interventional Radiology and Neuroradiology, University Hospital Essen, Hufelandstraße 55, 45147 Essen, Germany; 2 Institute for Artificial Intelligence in Medicine (IKIM), University Hospital Essen, Girardetstraße 2, 45131 Essen, Germany; 3 Department of Nuclear Medicine and German Cancer Consortium (DKTK), University Hospital Essen, University of Duisburg-Essen, Hufelandstraße 55, 45147 Essen, Germany; 4 Department of Nuclear Medicine, University Hospital Münster, University of Münster, Albert-Schweitzer-Campus 1, 48149 Münster, Germany

**Keywords:** Digital PET, PET, CT, Artificial intelligence, Low-count PET, Image post-reconstruction

## Abstract

**Purpose:**

Both digital positron emission tomography (PET) detector technologies and artificial intelligence based image post-reconstruction methods allow to reduce the PET acquisition time while maintaining diagnostic quality. The aim of this study was to acquire ultra-low-count fluorodeoxyglucose (FDG) ExtremePET images on a digital PET/computed tomography (CT) scanner at an acquisition time comparable to a CT scan and to generate synthetic full-dose PET images using an artificial neural network.

**Methods:**

This is a prospective, single-arm, single-center phase I/II imaging study. A total of 587 patients were included. For each patient, a standard and an ultra-low-count FDG PET/CT scan (whole-body acquisition time about 30 s) were acquired. A modified pix2pixHD deep-learning network was trained employing 387 data sets as training and 200 as test cohort. Three models (PET-only and PET/CT with or without group convolution) were compared. Detectability and quantification were evaluated.

**Results:**

The PET/CT input model with group convolution performed best regarding lesion signal recovery and was selected for detailed evaluation. Synthetic PET images were of high visual image quality; mean absolute lesion SUV_max_ (maximum standardized uptake value) difference was 1.5. Patient-based sensitivity and specificity for lesion detection were 79% and 100%, respectively. Not-detected lesions were of lower tracer uptake and lesion volume. In a matched-pair comparison, patient-based (lesion-based) detection rate was 89% (78%) for PERCIST (PET response criteria in solid tumors)-measurable and 36% (22%) for non PERCIST-measurable lesions.

**Conclusion:**

Lesion detectability and lesion quantification were promising in the context of extremely fast acquisition times. Possible application scenarios might include re-staging of late-stage cancer patients, in whom assessment of total tumor burden can be of higher relevance than detailed evaluation of small and low-uptake lesions.

**Supplementary Information:**

The online version contains supplementary material available at 10.1007/s00259-022-05901-x.

## Introduction

Both the recently introduced “digital” positron emission tomography (PET) / computed tomography (CT) systems and deep-learning based PET image post-processing tools have the potential to decrease the scanning time duration while maintaining clinically relevant diagnostic information. If current standard scan protocols are applied, the typical time required for a whole-body fluorodeoxyglucose (FDG) PET scan lies in the range of 20–45 min [1]. For reasons of patient comfort, particularly for anguished, dyspneic, or pediatric patients and for reasons of cost effectiveness, shorter acquisition times are desirable [2]. Using a maximum table speed velocity of a modern digital PET/CT scanner, acquisition times of 20 to 30 s per whole-body scan that match acquisition time of a CT scan are technically feasible. However, one of the major challenges remains the tradeoff between reducing the acquisition time and conserving clinical information, as time reductions consistently result in a significant deterioration of image quality [1].

Digital PET/CT systems use silicon-photomultipliers (SiPM) that exhibit an increased detector sensitivity resulting in a higher spatial resolution and coincidence time resolution when compared to previous-generation photomultiplier-tube-based systems [3–6]. A higher signal recovery increases the detectability of small and low-count lesions [5, 7–10]. In particular, low-count PET images reconstructed with time-of-flight (TOF) option benefit from the improved time resolution characteristics of SiPM-based PET [11]. Consequently, the acquisition time might be reduced without loss of clinically relevant information. Several phantom and clinical studies by our group and others demonstrated that, depending on the clinical question, a reduction in acquisition time up to a factor of $${\raise0.7ex\hbox{$1$} \!\mathord{\left/ {\vphantom {1 3}}\right.\kern-\nulldelimiterspace} \!\lower0.7ex\hbox{$3$}}$$ can be feasible for different radionuclides including ^18^F-FDG without image post-processing [1, 12–16].

Over the last several years, deep-learning networks have become increasingly valuable and potent tools within the field of medical imaging for various tasks [17]. Deep-learning models are actively employed in the analysis of CT, computed radiography (CR), and magnetic resonance imaging (MRI) scans for purposes including, but not limited to, diagnostics, tumor segmentations, and image post-reconstruction under low dose or undersampling conditions [18–21]. One field within the medical imaging domain that has recently sparked interest is medical image-to-image (I2I) translations. In this process, source domain images are transformed into synthetic images in a way that they adopt characteristics of target domain images [17, 22].

In recent years, a particular deep learning regime has shown to produce appealing results in I2I tasks, namely the Generative Adversarial Network (GAN) [23]. GANs are used for a variety of medical imaging I2I applications, such as contrast enhancement within CT scans, motion correction in MRI scans, PET to CT translations, and the de-noising of PET scans [17, 24]. To the group of GANs which have been proven to yield reliable results belong conditional GANs (cGANs) and especially the pix2pix [25] and its successor the pix2pixHD [26].

Several recent studies used artificial intelligence-based methods to enhance PET images acquired at low count rates (i.e., low acquisition times or low administered activities) [27–35]. However, to our best knowledge, none of these studies used a combination of data acquisition on a digital PET/CT system and GAN-based image post-processing for whole-body PET data. Moreover, many approaches were restricted to brain PET images [31–34], and in studies using whole-body data, the number of included patients was low [29, 35]. Additionally, acquisition times were longer than the maximum scan velocity of recent PET/CT scanners by a factor of at least 10 [27, 28]. Hence, the aim of this prospective phase I/II imaging study was to investigate the feasibility of acquiring maximum-speed ultra-short FDG ExtremePET images (scan time durations of about 30 s, > 33-fold reduced scan duration compared to clinical routine) on a digital PET/CT scanner and implementing a pix2pixHD network to recreate whole-body PET images (AI-ExtremePET) which are comparable to full acquisition time PET (FullTime-PET) scans. Several metrics including quantitative comparisons of PET signal recovery and manual image reading are evaluated to compare the physical image quality and the conservation of clinical information and lesion detectability among AI-ExtemePET and ground truth FullTime-PET images. We hypothesize that most of the diagnostically relevant information can be recovered from ExtremePET scans, whereas the quantitative evaluation of small and low count lesions might be challenging.

## Materials and methods

### Dataset

#### Patient cohort/ethics statement

This is a prospective, single-arm, single-center phase I/II imaging study. All patients who were referred for a clinical FDG PET/CT scan to the Department of Nuclear Medicine at the University Hospital of Essen, Germany, and who were scheduled for examination with a silicon-photomultiplier based PET/CT system between January 2020 and June 2020 were offered study participation in the order they appeared in clinical routine. Only patients < 18 years of age were excluded. A total of 587 patients were included in this prospective study (260 female patients and 327 male patients, mean age of 60.9 ± 13.2 and mean weight of 79.0 ± 18.7). The study was performed in accordance with the Declaration of Helsinki and approved by the local ethics committee (Ethics committee, University Duisburg-Essen, Faculty of Medicine, Ethics protocol number 20–9226-BO). Written informed consent was requested prior to enrollment.

#### FDG-PET/CT imaging

**Fig. 1:**
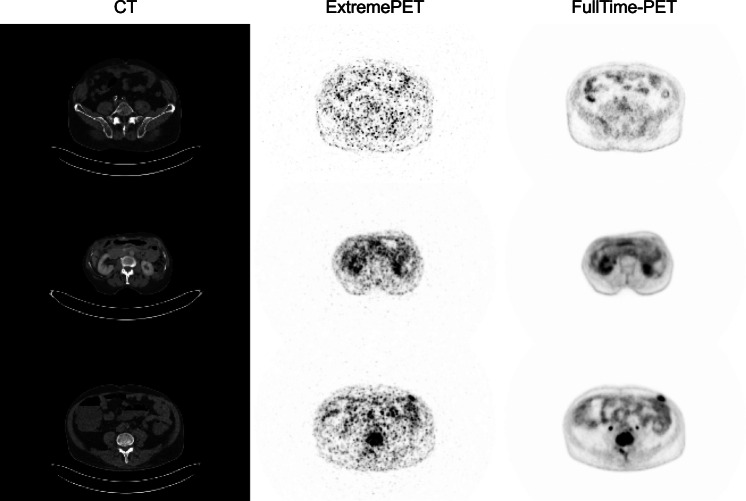
Collection of exemplary input pairs used within the training process. From left to right: **A**) CT, **B**) ExtremePET, and **C**) FullTime-PET

PET/CT data were acquired using a Biograph Vision 600 PET/CT System (Siemens Healthineers, Erlangen, Germany). PET/CT scans started with a CT scan in full-dose or low-dose technique according to the clinical routine protocol. Subsequently, reduced acquisition time PET data were first acquired in continuous bed motion mode using a table speed velocity of 50 mm/s (ExtremePET); the applied velocity was the fastest possible on the 26.3-cm field-of-view Biograph Vision PET/CT system. Normal acquisition time data were then acquired using a table speed velocity of 1.5 mm/s with an emphasis of the abdominal region by a reduced table speed velocity of 0.8 mm/s (FullTime-PET); in lung cancer patients instead of the abdominal region the thoracic region was emphasized. The mean ± SD applied activity was 327.8 ± 76.7 MBq of ^18^F-FDG. The mean ± SD interval between tracer application and start of PET scan was 74.6 ± 17.4 min. PET data were reconstructed using three-dimensional Poisson ordered-subsets expectation maximization with time-of-flight option (4 iterations 5 subsets, matrix size 220, voxel size 3.3 × 3.3 × 3.0 mm^3^, Gaussian filter 4 mm). Image datasets comprised a median number of 289 slices. Image examples are presented in Fig. 1.

#### Data preprocessing

Images extracted from two different methods, specifically PET and CT scans, were used as potential network inputs. Correspondingly, two different input settings were defined, namely PET and PET/CT.

For each method, 2.5-dimensional (2.5d) images were created using the axial slices. This is achieved by selecting a particular slice from the image volume, and then selecting the slices immediately prior and successive to the original slice. The first and last slices of each volume were ignored for the selection of central slices and only used to complete the 3-channel representation of the second and the penultimate slice, as, due to technical reasons in PET image acquisition, they contain mostly image noise and no clinically relevant information. Stacking results in a three-channel image; the generated 2.5d image output is a single image corresponding to the central slice that contains additional ​​information of the adjacent slides.

**Fig. 2:**
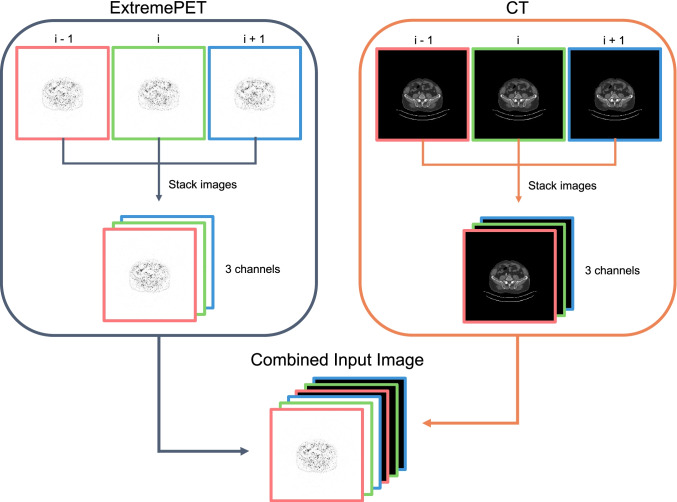
The used input slice pipeline for both input variations (PET-only and PET-CT). For each modality, a 3-channel image is generated including the previous and next slice. With this approach, more anatomical information is introduced to the 2D network

For the PET input setting, only 2.5d PET images were used as input for the pix2pixHD. For the PET/CT input setting, PET 2.5d and CT 2.5d images were combined on a channel axis to generate a six-channel image (Fig. 2). The six-channel image was then used as input. The intention behind the combined use of PET and CT scans is to investigate the hypothesis that CT images support the reconstruction of synthetic AI-ExtremePET images by providing more detailed anatomical information than the short acquisition time ExtremePET images.

All PET scans were transformed from Bq/ml to SUV units using the SUV Body Weight and normalized to a range of (0, 1) using a constant SUV maximum value of 50 to represent typical clinical conditions. Of note, information for lesions with SUV values > 50 could be lost. However, typical lesion SUV values in FDG PET imaging are much lower than 50 [36]. All CT scans were first resampled using the corresponding PET scan in order to match the resolution and the dimensions of the PET scans. The CT scans were then normalized to a range of (0, 1) using (− 1000, 3000) as the Hounsfield unit minimum and maximum. The resulting input samples used a resolution of 220 × 220 pixels and were then padded to a resolution of 224 × 224 pixels. Finally, the normalized slices were transformed from (0, 1) to (− 1, 1).

### Network architecture

General Adversarial Networks consist of two parts, a generator and a discriminator, in competition with each other. Within an image-to-image translation setting, the generator takes an input image from a source domain and generates a corresponding image with characteristics of the target domain. The discriminator then has to distinguish between real images and the images produced by the generator. The joint training process involves optimizing both the ability of the generator to produce images as similar as possible to the target domain as well as the ability of the discriminator to assign correct labels to sample images [25, 26].

For this study, we implemented a modified TensorFlow version of the pix2pixHD. The pix2pixHD is an extension of the pix2pix architecture, but, unlike the original pix2pix, uses two generators that operate with different resolutions in order to aggregate both local and global features of the image. This pair of generators constitutes a “coarse-to-fine” generator. The discriminator of the pix2pixHD is a multi-scale discriminator that itself employs three separate discriminators, each operating on a different image scale [25, 26].

The model was trained using a GAN and a feature-matching loss function in combination, as proposed by Wang et al. [26]. Additionally, we implemented an average pooling operation within the first downsample layers. For the first convolution in the generators’ encoders, we also added the option of a group convolution. This allows us to train models that process PET and CT images separately in the first convolution layer using 2 groups with 3 channels each. All models use an initial convolution filter size of 7 × 7 and an average pooling operation in the encoders. A batch size of four images was used during training, and the output channel was set to grayscale. A learning rate of 0.002 was used, and all models were trained with 100 epochs, with a learning rate decay starting after 50 epochs. A complete list of the used model settings is listed within the Supplemental Material (Table 3).

A total of three model configurations were used and trained: a model that uses only ExtremePET images (M1), an ExtremePET/CT model that uses both ExtremePET and CT images without group convolution (M2), and an ExtremePET/CT model that uses both ExtremePET and CT images with group convolution (M3). All model configurations were trained using a fivefold cross-validation (CV), and the final prediction for each model was aggregated using the mean overall CV model predictions.

All models used an image input size of 224 × 224. A total of 387 studies were used as training, and 200 studies were used as a test cohort.

### Evaluation methods

For this study, the evaluation process was performed in multiple parts. Firstly, all trained models were evaluated on the 200 test studies using the well-established I2I metrics Structural Similarity Index Measure (SSIM), Peak Signal to Noise Ratio (PSNR), and SUV-based Mean Absolute Error (MAE) [37–39]. For calculation of the scores, the voxel space outside the patient was excluded (masked with 0) using patient specific body masks. With this approach, we want to ensure that the score calculation is focused on the important region of interest, the body of the patient [40].

Secondly, 50 studies were randomly selected from the 200 test studies and manually examined by an experienced nuclear medicine physician. First, the human reader segmented all clinically relevant lesions visible within the FullTime-PETscan (excluding physiological tracer uptake). In addition, all detected lesions were separated into the following anatomical categories: bones, liver, lung, lymph nodes, and other. Determination of anatomical labels was performed with the assistance of a ​​software research prototype implementing a neural network (MICIIS, formerly MI Whole Body Analysis Suite, MIWBAS, Siemens Healthineers, Knoxville, TN, United States) [41]. The lesion segmentations were then used to evaluate and compare the SUV metrics SUV_mean_, SUV_max_, and SUV_peak_ for all models.

The synthetic AI-ExtremePET images of the best performing model (with respect to lesion SUV recovery) were then evaluated by the human reader to identify and segment all clinically relevant lesions (excluding physiological tracer uptake). All evaluations of synthetic AI-ExtremePET images were performed on a separate occasion from the evaluations of the original FullTime-PET scans to avoid bias due to prior knowledge. This leads to a total set of 50 Fulltime-PET segmentation masks and corresponding 50 AI-ExtremePET segmentation masks.

Lastly, the prepared segmentations were used to evaluate the lesion reconstruction quality of the best performing model. First, on the patient level, the detection of any lesion (dichotomous variable for each study) was compared among AI-ExtremePET and FullTimePET images. Next, to evaluate detection at the correct anatomical position, the given segmentation masks of the original and synthetic PETs were compared based on the Intersection over Union (IoU). In this analysis, a lesion is considered detected if the IoU is > 0. The IoU threshold is set to the proposed level because the compared masks are based on different PET images (original and synthetic). With this setting, we want to ensure that the evaluation is focused on the model’s ability to reconstruct lesions at the correct position without demanding perfect voxel matching. If more than one synthetic lesion candidate exists for an original lesion, the candidate with the highest IoU is selected. Detection at the correct anatomical position was evaluated on a patient, organ, and lesion level. Patient-based detection rate was defined as described above. On the organ level, the detection of any lesion in the specific organ (dichotomous variable for each organ and each study) was compared among AI-ExtremePET and FullTimePET images. Lesion-based detection rate includes all detected lesions in the FullTimePET images. For these evaluations, additionally, all lesions were separated into two groups: PERCIST-measurable and non PERCIST-measurable. The PERCIST group contains all lesions which satisfy the PERCIST criteria (SUV_peak_ > 1.5 × mean(SUV_liver_) + 2 × std(SUV_liver_)) [42, 43]. Detection rates at the correct anatomical position were then separately calculated for PERCIST-measurable and non PERCIST-measurable lesions.

### Statistics

For statistical analysis, we used the two-sided nonparametric Mann–Whitney *U* [44] test using the python package scipy [45].

## Results

Synthetic PET images for all three models were of high visual image quality and showed significant improvements in SSIM, PSNR, and MAE compared to the ultra-short ExtemePET images (using the FullTime PET as ground truth, details in Supplemental Material, Fig. 6). To select a model for detailed evaluation, first, a comparison of the lesion SUV reconstruction quality was performed. For this approach, the lesion masks (containing all lesions that were detected by a human reader in the FullTime-PET) are used to compare SUV_mean_, SUV_max_, and SUV_peak_ differences (between FullTime-PET and AI-ExtremePET images) for each model. Of 50 manually evaluated cases, 33 contained lesions (66%). Within those 33 cases, 298 lesions were detected by the human reader. The organ-based SUV level comparison is presented in Table 1.

**Table 1:** Differences of the SUV_mean_, SUV_max_, and SUV_peak_ values between the FullTime-PET and the AI-ExtremePET images for each model based on the original lesion segmentation mask. The SUV_mean_ is calculated based on the mean SUV values present within the lesion mask. The SUV_max_ is calculated using the maximum SUV value for each lesion mask. The SUV_peak_ is calculated using a 1-cm^3^ sphere around the maximum voxel within a lesion mask

Body part	M1			M2			M3		
	SUV_mean_	SUV_max_	SUV_peak_	SUV_mean_	SUV_max_	SUV_peak_	SUV_mean_	SUV_max_	SUV_peak_
All	1.01 ± 1.43	1.87 ± 2.28	0.76 ± 1.60	2.11 ± 1.65	3.45 ± 2.60	1.94 ± 1.66	0.91 ± 1.54	1.50 ± 2.46	0.57 ± 1.74
Bones	1.02 ± 0.79	2.15 ± 1.86	0.72 ± 0.96	2.84 ± 1.19	4.84 ± 2.21	2.57 ± 1.39	1.05 ± 1.05	1.90 ± 2.13	0.63 ± 1.26
Liver	1.26 ± 0.50	2.62 ± 1.54	1.41 ± 0.76	2.09 ± 0.87	3.43 ± 1.95	2.31 ± 1.19	1.07 ± 0.62	2.09 ± 1.98	1.01 ± 0.98
Lung	1.16 ± 1.04	1.89 ± 1.63	0.82 ± 0.88	2.08 ± 1.01	3.05 ± 1.67	1.81 ± 1.09	0.87 ± 0.99	1.04 ± 1.94	0.39 ± 1.19
Lymph nodes	1.21 ± 0.86	1.93 ± 1.53	0.81 ± 0.87	2.01 ± 1.14	3.11 ± 1.69	1.83 ± 1.11	1.03 ± 1.00	1.38 ± 1.73	0.54 ± 0.89
Other	2.51 ± 3.73	3.78 ± 5.15	2.33 ± 4.48	3.06 ± 3.05	4.63 ± 3.80	2.94 ± 3.09	2.34 ± 3.84	3.45 ± 5.36	2.09 ± 4.69

**Fig. 3:**
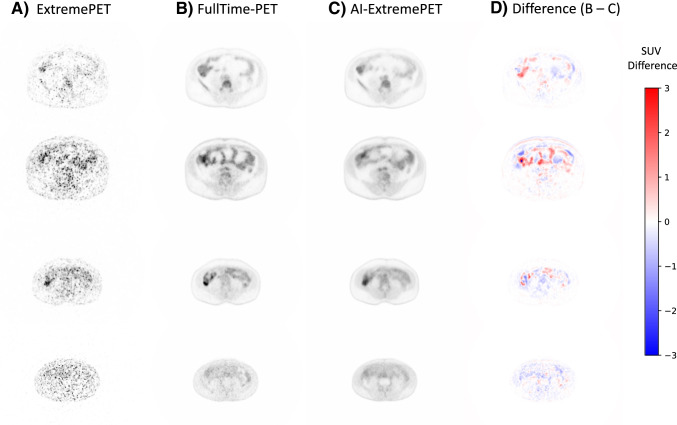
Collage of exemplary reconstructions from model M3 within the following order (left to right): Input (**A**), target (**B**), prediction (**C**), and difference map (**D**). The difference map is based on the subtraction of the original FullTime-PET with the AI-ExtremePET. Red spots indicate that the reconstructed intensity was lower than in the FullTime-PET, and blue spots indicate that the reconstructed intensity was higher than in the AI-ExtremePET

The results show that model M3 performs best for lesion evaluation. We therefore used the M3 AI-ExtremePET images for further evaluation. Figure 3 depicts reconstructed slices from model M3, including a difference map (FullTime-PET versus AI-ExtemePET) for multiple cases.

**Fig. 4:**
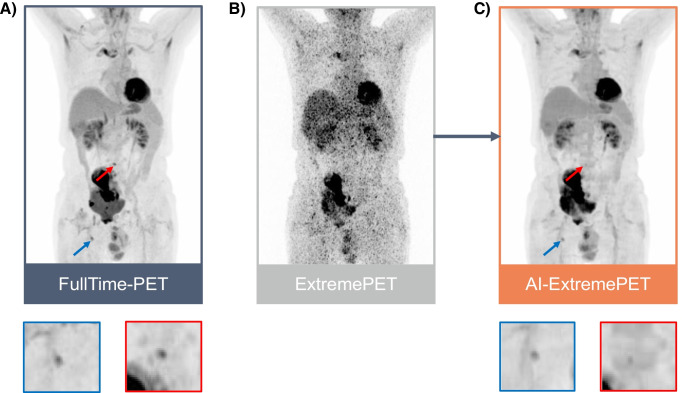
Exemplary representation of maximum-intensity-projections (anterior view) of the ground truth FullTime-PET (**A**), the ExtremePET (**B**), and the M3 AI-ExtremePET (**C**). In the FullTime-PET, two lesions were detected (red and blue arrows), of which one (blue) was restored in the AI-ExtremePET. In the ExtremePET, no lesions were detected. At the bottom, magnified views of both lesions are presented (the colors of their borders correspond to the colors of the respective arrows in the original images)

Patient-based sensitivity and specificity for lesion detection were 79% and 100%, respectively. An exemplary patient image showing a correctly detected and a missed lesion in the AI-ExtremePET images is presented in Fig. 4.

Next, we separately calculated patient-, organ-, and lesion-based detection rates in a matched pair approach. The patient-based detection rate was 79% for all lesions. Lesions that were not detected in the AI-ExtremePET images were of smaller volume (median volume: 1.0 ml versus 2.7 ml, *p* = 0.06) and tracer uptake in the ExtremePET than correctly detected lesions (median SUV_peak_: 3.1 versus 4.9, *p* = 0.05; median SUV_mean_: 2.7 versus 4.6, *p* < 0.001). We, therefore, split the data according to the clinically established PERCIST [43] criteria. From 298 detected lesions, 229 were PERCIST-measurable. The patient-based detection rate was 89% regarding only PERCIST-measurable lesions, and 36% regarding only non PERCIST-measurable lesions. The lesion-based detection rate was 65% for all lesions, 78% for PERCIST-measurable lesions, and 22% for non PERCIST-measurable lesions. Detailed organ-based detection statistics (indicating detection rates, mean SUV levels, and lesion volumes) are presented in Table 2.

**Table 2:** Detailed lesion-based detection characteristics. SUV values were estimated in the ExtremePET images and based on the original lesion masks

Body part	Lesion cohort	Detected	n	Volume (ml)	SUV_max_	SUV_mean_	SUV_peak_	Detection rate
All	PERCIST	True	178	8.56 ± 21.13	12.87 ± 7.22	5.78 ± 2.98	6.62 ± 4.49	0.78
		False	51	1.69 ± 2.64	9.75 ± 5.23	4.06 ± 1.35	4.15 ± 1.33	
	Other	True	15	5.69 ± 9.72	7.62 ± 2.86	2.87 ± 0.98	2.90 ± 0.85	0.22
		False	54	1.87 ± 2.55	5.82 ± 2.73	2.07 ± 0.65	2.35 ± 0.92	
Bones	PERCIST	True	119	3.60 ± 3.27	12.03 ± 5.22	5.65 ± 2.33	6.07 ± 2.94	0.84
		False	23	2.15 ± 3.37	8.07 ± 2.23	4.29 ± 1.11	3.79 ± 1.01	
	Other	True	5	0.85 ± 0.65	5.89 ± 2.74	3.19 ± 1.35	2.46 ± 0.70	0.42
		False	7	1.10 ± 1.36	5.36 ± 0.93	2.13 ± 0.35	2.67 ± 1.09	
Liver	PERCIST	True	5	32.74 ± 33.29	12.93 ± 2.47	4.94 ± 0.74	6.20 ± 0.94	0.83
		False	1	1.63	7.87	4.01	4.27	
	Other	True	-	-	-	-	-	0.0
		False	3	1.61 ± 2.79	8.73 ± 2.52	2.74 ± 0.29	3.48 ± 0.44	
Lung	PERCIST	True	21	5.84 ± 11.18	12.34 ± 8.41	5.25 ± 3.57	6.01 ± 5.33	0.88
		False	3	1.47 ± 1.51	6.97 ± 2.18	3.51 ± 1.44	3.50 ± 0.94	
	Other	True	3	2.35 ± 1.61	7.58 ± 3.33	2.21 ± 1.12	2.30 ± 0.87	0.23
		False	10	1.16 ± 1.26	5.06 ± 3.79	1.70 ± 0.81	1.58 ± 0.67	
Lymph nodes	PERCIST	True	27	11.81 ± 17.64	13.87 ± 7.36	5.96 ± 3.78	7.40 ± 4.88	0.63
		False	16	1.54 ± 2.08	10.83 ± 6.20	4.10 ± 1.68	4.41 ± 1.72	
	Other	True	6	12.19 ± 13.34	8.81 ± 2.71	2.79 ± 0.46	3.56 ± 0.62	0.18
		False	27	2.68 ± 3.17	6.04 ± 2.42	2.20 ± 0.54	2.47 ± 0.77	
Other	PERCIST	True	6	81.77 ± 69.68	26.97 ± 19.27	10.10 ± 6.02	16.54 ± 11.88	0.43
		False	8	0.75 ± 1.50	13.70 ± 7.89	3.54 ± 1.35	4.91 ± 1.22	
	Other	True	1	0.88	9.25	3.79	2.89	0.12
		False	7	0.64 ± 0.98	5.28 ± 3.13	1.76 ± 0.86	2.17 ± 1.01	

Moreover, lesion volume differences between AI-Extreme-PET and FullTime-PET images were lower for PERCIST-measurable in comparison to non PERCIST-measurable lesions, whereas the IoU was higher (Supplemental Material, Table 4). This, additionally, indicates an improved reproduction of PERCIST-measurable lesions.

**Fig. 5:**
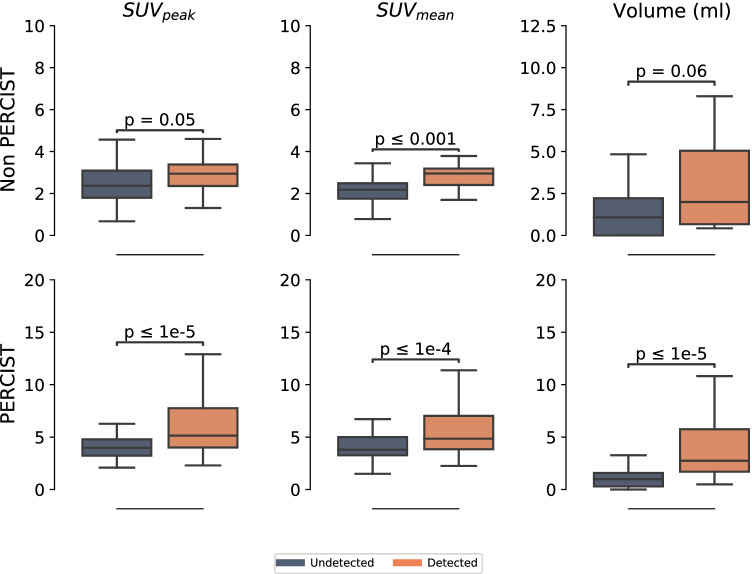
Distribution of the ExtremePET SUV_peak_, SUV_mean_, and lesion volume for non PERCIST (first row) and only PERCIST (second row) lesions with an additional statistical significance test (Mann–Whitney *U*). The bar in each box represents the median

PERCIST-measurable lesions that were not detected in the AI-ExtremePET images were of significantly smaller volume (median volume: 1.0 ml versus 2.7 ml, *p* < 0.0001) and tracer uptake (median SUV_peak_: 4.0 versus 5.1, *p* < 0.0001; median SUV_mean_: 3.8 versus 4.9, *p* < 0.0001) in the ExtremePET than correctly detected lesions (Fig. 5). For non PERCIST-measurable lesions, not detected lesions were of significantly smaller tracer uptake (median SUV_peak_: 2.4 versus 3.0, *p* = 0.05; median SUV_mean_: 2.2 versus 3.0, *p* < 0.001) but showed no significant difference in lesion volume (median volume: 1.1 ml versus 2.0 ml, *p* = 0.06).

## Discussion

In this study, we demonstrated the use of a pix2pixHD network to generate synthetic full acquisition time PET images from 33-fold reduced acquisition time ExtremePET images that were exclusively acquired on a “digital” silicon-photomultiplier based PET/CT scanner. Only few previous approaches have been described that use GANs for enhancement of low-count whole-body PET images [27, 28, 46]. Some previously published approaches were restricted to brain PET imaging [17, 31–34, 47], which is an easier process due to the limited anatomical variance compared to whole-body images [35]. In the following, we intend to evaluate the results of this study in the context of whole-body imaging.

Some approaches for whole-body low-count imaging enhancement do not implement GANs [28, 48, 49]. Compared to the few previous works that use GANs on whole-body PET images, in our study the acquisition time was shorter by about one magnitude, and the number of included patients was significantly larger. For example, Lei et al. use a CycleGAN for enhancement of 25 whole-body eightfold reduced PET data sets acquired on a conventional PET scanner [46]. Sanaat et al. implemented a ResNET and a CycleGAN and included 85 conventional sevenfold reduced PET data sets [27]. No previous study focused on the benefits of a digital PET scanner for low-count PET imaging, and no study that used a digital scanner implemented a GAN. Kaplan and Zhu used an estimator and an adversarial discriminator network on tenfold reduced digital PET data but included only 2 patients [49]. Most recently, Chaudhari et al. applied a commercially available encoder-decoder U-Net based solution on 50 fourfold reduced PET data sets that were only in parts acquired on a digital scanner [28]. The improved detector sensitivity, time and spatial resolution, and noise characteristics [3, 5, 6, 11] of SiPM-based PET provide the basis for acquiring ultra-low-count PET data with a whole-body scan time comparable to a CT scan.

In this proof-of-concept approach, we used the maximum possible PET scan velocity of a latest-generation digital PET/CT scanner. A visual evaluation revealed the technical feasibility to generate PET images of high visual image quality from noisy ultra-short acquisition time data (example in Fig. 5). A preserved high visual image quality is the prerequisite for further evaluation in terms of quantification and detectability, and a first relevant finding since the acquisition time in this study was in the range of a standard CT scan and, thus, significantly shorter than in previously published studies. Next, we compared SSIM, PSNR, and MAE, commonly applied criteria for assessment of quantification performance [27], among three different models using PET data only, PET/CT data without group convolution, or PET/CT data with group convolution in comparison to the ground truth full acquisition time images. The results were excellent for all three models and significantly improved compared to the ExtremePET images without post-reconstruction (Fig. 3). Next, we analyzed SUV differences for all lesions that were detected and segmented by a human reader in the ground truth FullTime PET images. This analysis showed best performance for the combined input model (M3) with group convolution (Table 1). We therefore selected this model for detailed evaluation. In most previous works, only PET data were used as input [27–29, 48, 49]. However, our results are in line with a PET/MRI study that describes benefits by simultaneous input of PET and MRI images for enhancement of ultra-low-dose PET images in children [35].

The detailed lesion quantification analysis for all lesions that were detected in the ground truth images revealed a mean ± SD absolute SUV_max_ difference of 1.5 ± 2.5 and a mean ± SD absolute SUV_mean_ difference of 0.9 ± 1.6. These are appealing results in the context of the extremely short acquisition time. For previous approaches, using longer acquisition times, lower SUV differences were described. Chaudhari et al. report a lesion mean SUV difference of approximately zero (95%-confidence limit of 1.8 for the SUVmax of the lesions) [28]. Sanaat et al. indicate a mean SUV_max_ difference of − 0.01 [27]. However, slight SUV differences, as in our study, do not influence patient management in most clinical circumstances, as clinical image reading in most cases does not depend on exact lesion quantification but on detectability of lesions with increased tracer uptake. The reason for the reduced SUV reproduction in our account approach is most probably by a factor of ≥ 10 reduced acquisition time in our approach and not a structural problem of the applied GAN. With regard to the ultra-low-count PET data we used, deviations in quantification are expectable, and exact quantification was not an aim of this study.

More than lesion quantification, lesion detectability is decisive to assess the possible value of ultra-short acquisition time PET images in a clinical context, as missed lesions can be of big influence on patient management. The patient-based analysis revealed a sensitivity of 79% and specificity of 100%; no patients were classified as false-positive. This is a relevant finding, as misclassification of patients as false-positive could have consequences to further patient/therapy management. Chaudari et al. reached a patient-level sensitivity of 94% and specificity of 98%, Sanaat et al. do not report a patient-based analysis but a lesion-based sensitivity of 97% [27]. The detailed analysis showed that missed lesions were of lower tracer uptake and lesion volume in the input ExtremePET images. To evaluate the clinical usability, we, therefore, performed additional separate analyses for PERCIST-measurable and non PERCIST-measurable lesions in a matched-pair approach and calculated patient-based, organ-based and lesion-based detection rates. These were largely increased for PERCIST-measurable lesions (for example, 89% versus 36% on the patient level and 78% versus 22% on the lesion level, details in Table 2). For PERCIST-measurable lesions not detected lesions were of significantly smaller tracer uptake (SUV_mean_) and volume, whereas for non PERCIST-measurable lesions only the difference in tracer uptake was significant (Fig. 5).

As the reproduction of lesions with low tracer uptake and small lesion volume remains challenging, the clinical applicability of AI-ExtremePET images is limited. As detectability was restricted for small lesions with low tracer uptake, primary staging and investigation of patients in the early phase of disease will probably be problematic. The analysis of PERCIST-measurable lesions showed an improved detectability for larger lesions of higher tracer uptake. Moreover, for PERCIST-measurable lesions the volume reproduction was improved (Supplemental Material, Table 4). Therefore, a possible setting for a clinical use might be follow-up of metastatic cancer patients in whom an evaluation of total tumor burden (with, potentially, high tracer uptake) is of larger clinical relevance than detection of single lesions. In this context, typically pain-stricken patients with high tumor burden could benefit from a short acquisition time. Reduced motion artifacts can be an additional advantage of short emission time PET scans [28].

To assess whether the AI-ExtremePET technique is suitable for follow-up staging, future studies could investigate reproducibility of quantitative measures and metabolic tumor volumes. Typically, test–retest SUV deviation in FDG PET scans is about ± 20% [50]. A detailed investigation would require two separate PET scans in short temporal distance and is, therefore, not possible using the data set of this study. Moreover, future studies could evaluate patients with a follow-up scan to investigate whether AI-ExtremePET images can be used for oncological response assessment.

For clinical applications in which a characterization of single lesions is decisive (e.g., initial staging or detection of primary tumor), accuracy of lesion quantification and detectability must be improved. Future studies might focus on probing different acquisition times to investigate the optimal tradeoff between scan duration and conservation of clinical information. Since previous studies, which show better results, use substantially lower reductions in acquisition time [27, 28], an investigation of the acquisition time range between their approach and ours could be promising. For example, an extension of the ExtremePET acquisition time by a factor of 2–5 could be investigated. However, an acquisition time optimization was beyond the protocol of this prospective study in which the fastest possible acquisition time of a current-generation digital PET/CT scanner was evaluated. Moreover, future approaches might use data acquired on total-body PET/CT scanners that exhibit improved counting statistics and might allow for even shorter acquisition times than standard digital PET/CT scanners while maintaining acceptable image quality [50]. However, until now, total-body PET scanners are expensive and have not widespreadly been introduced. As reductions in acquisition time below the scan duration that was applied in this study might not be more beneficial in daily practice, deep-learning based image enhancement might in future be used for total-body PET data to reduce the administered activity. A reduced acquisition time can be used as a surrogate for a reduced administered tracer activity, as these values, in a first approach, correlate linearly [16, 51].

In our study, ExtremePET data were used that were acquired in a separate ultra-short scan, whereas most previous studies use simulated short acquisition time data that are created by undersampling of original list-mode data sets [29, 35, 48, 49]. Sanaat et al. also use separately acquired low-dose PET data [27]. Chaudhari et al. describe a multicenter study that uses undersampled data from one center and separately acquired data sets from two centers [28]. The usage of real data is beneficial, since larger image noise in short acquisition time data sets might not be fully reproduced by undersampling [27, 51]. On the other hand, the separate PET data acquisition contributes to a further main limitation of the study. Possible deviations in spatial pairing between FullTime-PET and ExtremePET images due to patient motion might impair the reconstruction quality of the applied Pix2PixHD network. However, the ExtremePET scan was started directly after the FullTime-PET scan using the same scan area and patient position; this ensures a good co-registration between both image data sets. Moreover, most clinically relevant lesions are located in the trunk for which motion is low. Therefore, the benefits of separate PET acquisitions justify the limitations of spatial deviations.

Besides the Pix2PixHD that was used in this study, other neural network architectures could be used for I2I translations of PET images. For example, a cycleGAN setting could be used, which would tackle the problem of the spatial alignment of the different image types since the CycleGAN is used for unpaired image to image tasks [52]. Another future possibility might be the application of a transformer-based CycleGAN. These networks may be less susceptible to variations in spatial pairing and could therefore be promising for PET images, which are of lower resolution and higher image noise than CT or MRI images. A comparison of different networks could be the subject of future studies.

The study is affected by further limitations. Only PET/CT data from a single center and a single PET/CT scanner were included in the evaluation. For generalizability, a multicenter study using digital PET/CT scanners from different vendors should be performed. However, generalizability was not an aim of this proof-of-concept study, as first further steps of improvement and validation are necessary. In addition, the trained network only used stacked 2D axial slices within a 2.5D approach which could be updated within further studies to a 3D approach. An attention-weighted loss function could be used to emphasize the most significant body parts [32, 33]. Furthermore, the study was only performed for ^18^F-FDG PET/CT imaging. Future studies could cover different PET tracers. Previous approaches were described for brain PET imaging using ^18^F-Florbetaben [34], whereas for DOTATATE/DOTATOC and PSMA PET, to our best knowledge, no deep learning models to enhance low count images have been described.

## Conclusion

A combination of digital PET/CT and artificial intelligence-based image post-reconstruction allows the generation of high quality images from PET data that were acquired as fast as CT scans. Detectability (79% on a patient level) and lesion quantification revealed promising results; lesion tracer uptake and volume were lower for not-detected lesions. In the current form, the number of missed lesions still prevents a broad clinical use, but the approach could be applied in late-stage cancer patients to monitor total tumor burden. Future studies investigating ultra-fast PET imaging are warranted.

## Supplementary Information

Below is the link to the electronic supplementary material.

Supplementary file1 (DOCX 65.1 KB)

## Data Availability

The datasets generated during and/or analyzed during the current study are available from the corresponding author on reasonable request.
