# Supplementary material for: Artificial intelligence guided enhancement of digital PET: scans as fast as CT?
Source: Eur J Nucl Med Mol Imaging. 2022 Jul 29;49(13):4503–15. doi: 10.1007/s00259-022-05901-x (PMC9606065; doi:10.1007/s00259-022-05901-x)
Supplement: Supplementary file 1 — Supplementary file1 (DOCX 65.1 KB) [file 259_2022_5901_MOESM1_ESM.docx]

# Supplemental Material


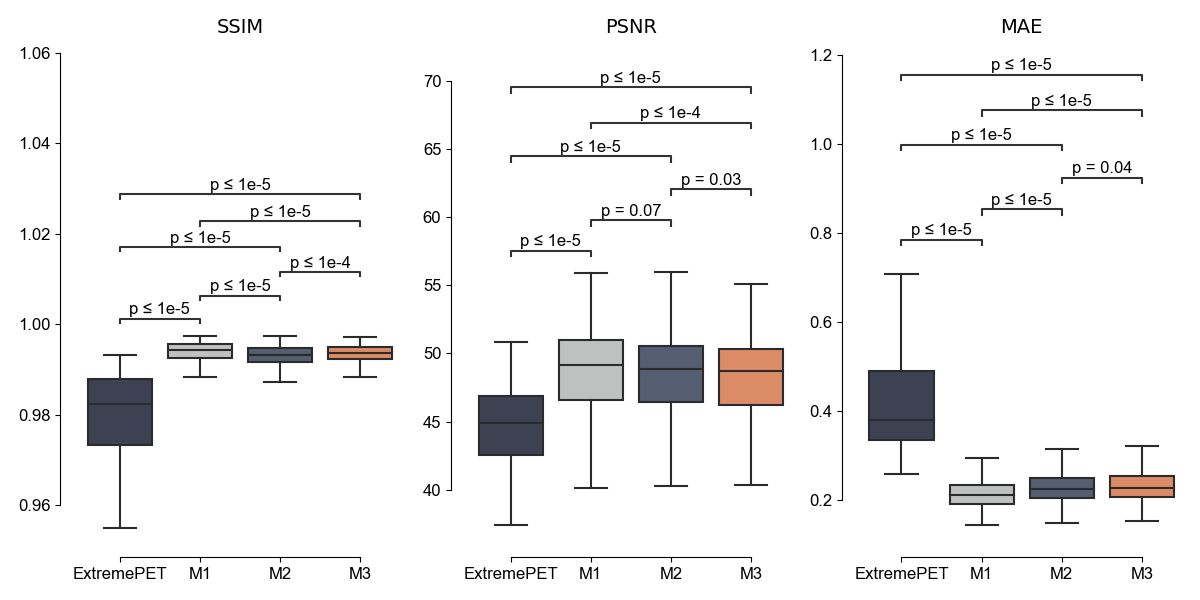


**Fig.** 6: Comparison of the I2I metrics SSIM, PSNR, and MAE using a boxplot visualization. The ExtremePET (baseline) scans as well as the three distinct models (M1, M2, and M3) were compared to the FullTime PET using a Mann-Whitney-U test. All three models perform significantly better than the baseline ExtremePET scan regarding the compared I2I metrics. In addition, all models were compared against each other for each metric. The scores were calculated using a body mask for each patient.

#

| Parameter | M1 | M2 | M3 |
| --- | --- | --- | --- |
| Input Type | PET | PET/CT | PET/CT |
| Input Channel | 3 | 6 | 6 |
| Output Channel | 1 | * | * |
| Input Size | 224x224 | * | * |
| Generator Architecture | U-NET | * | * |
| Generator Type | Local Enhancer | * | * |
| Base Filters | 64 | * | * |
| Initial Conv Size | 7x7 | * | * |
| Initial Conv Downsampling | AVG-Pooling | * | * |
| Group Convolution | False | False | True |
| Number Downsample Blocks | 4 | * | * |
| Number ResUnits Global | 9 | * | * |
| Number of ResUnits Local | 3 | * | * |
| Upsampling Operation | Transpose Convolution | * | * |
| Number of Discriminators | 2 | * | * |
| Discriminator initial Conv Filters | 64 | * | * |
| Discriminator Layers | 3 | * | * |
| GAN Loss | LSGAN | * | * |
| Feature Matching Loss | L1 | * | * |
| Feature Matching Loss Weight | 10 | * | * |
| Perceptual Loss Weight | 0.15 | * | * |
| Learning Rate | 0.002 | * | * |
| Momentum | 0.5 | * | * |
| Epochs | 100 | * | * |
| Learning Rate Decay  (Start / Duration) | 50/50 | * | * |
| Batch Size | 4 | * | * |

**Table** 3: List of the used hyperparameters and network settings for all three models. The * indicates that the value remains unchanged for subsequent models.

| Lesion Cohort | IoU | Volume Difference | SUV_peak_ Difference |
| --- | --- | --- | --- |
| PERCIST | 0.4 ± 0.2 | 0.35 ± 9.11 | 0.59 ± 2.19 |
| Other | 0.19 ± 0.16 | -3.41 ± 14.43 | 0.13 ± 0.76 |

**Table** 4: Mean and standard deviation evaluation for detected PERCIST as well as Non-PERCIST (other) lesions regarding the difference in lesion volume, SUV_peak_ (original vs synthetic) and the respective IoU for the model M3.
